# Polycomb-group protein SlMSI1 represses the expression of fruit-ripening genes to prolong shelf life in tomato

**DOI:** 10.1038/srep31806

**Published:** 2016-08-25

**Authors:** Dan-Dan Liu, Li-Jie Zhou, Mou-Jing Fang, Qing-Long Dong, Xiu-Hong An, Chun-Xiang You, Yu-Jin Hao

**Affiliations:** 1 National Key Laboratory of Crop Biology, Shandong Agricultural University, Tai-An, Shandong 271018, China; 2 College of Horticulture Science and Engineering, Shandong Agricultural University, Tai-An, Shandong 271018, China; 3 College of Agriculture, Yunnan University, Kunming, Yunnan 650091, China

## Abstract

Polycomb-group (PcG) protein MULTICOPY SUPPRESSOR OF IRA1 (MSI1) protein is an evolutionarily conserved developmental suppressor and plays a crucial role in regulating epigenetic modulations. However, the potential role and function of MSI1 in fleshy fruits remain unknown. In this study, *SlMSI1* was cloned and transformed into tomato to explore its function. The quantitative real-time PCR results showed that *SlMSI1* was highly expressed in flowers and fruits and that its transcript and protein levels were significantly decreased in fruits after the breaker stage. Additionally, *SlMSI1-*overexpressing transgenic tomatoes displayed abnormal non-ripening fruit formation, whereas its suppression promoted fruit ripening in transgenic tomatoes. Quantitative real-time PCR assays also showed that *RIN* and its regulons were decreased in *SlMSI1* overexpression transgenic tomato fruits. Furthermore, RNA-seq analysis demonstrated that *SlMSI1* inhibits fruit ripening by negatively regulating a large set of fruit-ripening genes in addition to *RIN* and its regulons. Finally, genetic manipulation of *SlMSI1* and *RIN* successfully prolonged the fruit shelf life by regulating the fruit-ripening genes in tomato. Our findings reveal a novel regulatory function of *SlMSI1* in fruit ripening and provide a new regulator that may be useful for genetic engineering and modification of fruit shelf life.

Polycomb-group (PcG) proteins, which were first identified in *Drosophila melanogaster*, are evolutionarily conserved developmental suppressors and play a crucial role in regulating epigenetic modulations in plant and animal species^1^,^2^,^3^. These PcG proteins form distinct complexes to control vegetative and reproductive development and play important roles in phase transitions during development, cell fate determination and cellular differentiation by repressing sets of genes that regulate either proliferation or differentiation in plants^4^,^5^,^6^. The well-studied PcG complexes include the polycomb repressive complex 2 (PRC2) and PRC1, which are thought to act in a sequential manner to stably maintain gene repression^4^. PRC2 induces histone H3 trimethylation of lysine 27 (H3K27me3), which is sequentially read by PRC1 that catalyzes H2A monoubiquitination (H2Aub1) to stably repress target genes expression^7^. PRC1 and PRC2 complexes play a global role in mediating gene regulation networks during plant development.

Numerous PcG proteins have been purified. The components of PRC1 are considerably different between animals and plants, whereas the components of PRC2 are evolutionarily conserved. In *Arabidopsis*, there is only one PRC1 complex, which consists of LIKE HETEROCHROMATIN PROTEIN1 (LHP1), AtRING1A/AtRING1B, AtBMI1A/AtBMI1B, and EMBRYONIC FLOWER1 (EMF1) [probably VERTILIZATION 1 (VRN1)]. PRC1 functions to maintain H3K27me3, perform histone H2A monoubiquitination (H2Aub1), and/or condense chromatin to inhibit transcription^8^. The PRC2 complex in *Drosophila* is composed of four core elements: histone methyltransferase enhancer of zeste (E(z)), which has three homologous proteins, CURLY LEAF (CLF), SWINGER (SWN), MEDEA (MEA), in *Arabidopsis*; Suppressor of zeste 12 (Su(z)12), which has three homologous proteins, EMBRYONIC FLOWER 2 (EMF2), FERTILIZATION-INDEPDENT SEED 2 (FIS2) and VRN2, in *Arabidopsis*; extra sex comb protein (Esc), which has only one homologous protein, FERTILIZATION-INDEPENDENT ENDOSPERM (FIE), in *Arabidopsis*; and core protein p55, which has five homologous proteins, i.e., MULTICOPY SUPPRESSOR OF IRA 1–5 (MSI1-5), in *Arabidopsis*^9^. In addition, these proteins form three types of PRC2 complexes, i.e., FERTILIZATION INDEPENDENT SEED (FIS)-PRC2, VRN-PRC2, and EMF-PRC2. The FIS-PRC2 complex contains FIS1/MEA, FIS2, FIS3/MEA, and MSI1, which functions in regulating female gametophyte and endosperm development^10^,^11^. The VRN-PRC2 complex is composed of VRN2, FIS3/FIE, CLF (or SWN) and MSI1 and accelerates flowering in response to prolonged exposure to cold^12^,^13^. The EMF-PRC2 complex is composed of EMF2, FIS3/FIE, CLF (or SWN) and MSI1 and controls vegetative development and the transition to flowering^9^,^14^.

PcG proteins are evolutionarily conserved developmental suppressors and play a crucial role in regulating epigenetic modulations, i.e., DNA methylation and histone methylation, in plant and animal species^1^,^3^. In tomato, a global methylome analysis demonstrated that DNA methylation has crucial regulatory roles in tomato fruit development and maturation by controlling the timing of the ripening process^15^,^16^,^17^. The tomato is a popular model plant for studying fruit ripening^18^. In recent decades, investigations have focused on the identification of spontaneous ripening-deficient mutants and characterization of the increasing number of known ripening-related transcription factors (TFs)^19^,^20^. Of these known TFs, the MADS-box protein RIN (*RIPENING INHIBITOR*) appears to be a master regulator that is critical for the transcriptional regulation of ripening initiation and progression by directly binding to a conserved *cis*-element known as the C-(A/T)-rich-G (CArG) box in the promoter regions of itself and various other ripening genes^21^,^22^,^23^. Studies on *RIN* and its regulon have elucidated the complicated regulatory network of fruit ripening. Identification of the tomato epiallele gene *CNR (COLOURLESS NON-RIPENING*) has provided strong evidence for the possible role of epigenetic processes in fruit ripening^24^. Recently, increasing evidence has indicated that epigenetic modulations are involved in the control of fruit ripening. Some PcG genes, such as *SlEZ1*^25^ and *SlVIN3*^26^, have been found to regulate flower/fruit development and floral organ differentiation in tomato, and *SlEZ2*^27^ has been shown to function in fruit development and ripening.

In *Arabidopsis*, MSI1 is an indispensable component of the PRC2 complex. A mutation in *MSI1* affects vegetative development, transition to flowering and seed formation^6^,^28^. In tomato, it has been shown that SlMSI1 binds to a 65 kD protein during fruit ripening^29^. However, its function in fleshy fruits is largely unknown. In this study, *SlMSI1* was identified as having a crucial function in fruit ripening. It repressed the expression of *RIN* and other fruit ripening genes. Additionally, the utilization of SlMSI1-mediated biotechnology in the genetic manipulation of fruit shelf life was investigated and discussed.

## Results

### SlMSI1 transcript and protein levels decrease with fruit ripening in tomato

**Figure 1:**
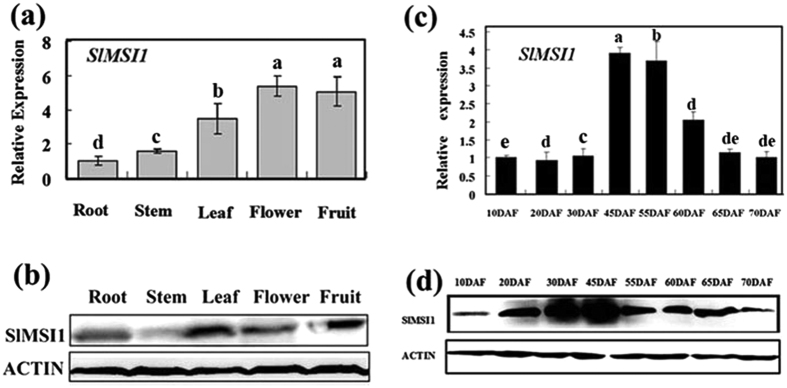
Expression and protein profiles of SlMSI1 in tomato. (**a,b**) Expression and protein level analysis of SlMSI1 in different organs of tomato. (**c,d**) Transcript and protein level analysis of SlMSI1 during fruit development. SlMSI1 RNA and protein samples were derived from the same experiment, and electrophoretic gels and protein blots were processed in parallel. The same letter in the same growing season means no significant differences among three biological replicates (P < 0.05). Error bars represent SE.

Based on the sequence in the tomato genome (https://solgenomics.net/organism/Solanum_lycopersicum/genome), full-length cDNA of *SlMSI1 (Solyc01g104510.2.1*) was cloned from *Alisa Craig* tomato. To explore its tissue-specific expression profile, quantitative real-time PCR (qRT-PCR) and western blot assays were performed to determine the temporal and spatial patterns of SlMSI1 transcripts/proteins in tomato roots, stems, leaves, flowers and fruits at different ripening stages. The results showed that SlMSI1 was constitutively accumulated in all tested organs, especially in the flower and fruit (Fig. 1A,B), thus suggesting a potential role in reproductive development.

For fruits, the entire ripening process was divided into eight stages, which were indicated as the day after anthesis (dpa). The SlMSI1 transcript and protein levels were found to vary with fruit development and ripening (Fig. 1C,D). Both levels were very low during the early stages of fruit development and then dramatically increased up to a maximum level at 45 dpa. Subsequently, SlMSI1 transcript and protein levels gradually decreased with fruit ripening, especially after the breaker stages (Fig. 1C,D), suggesting that *SlMSI1* is involved in the regulation of fruit ripening in tomato.

### SlMSI1 affects fruit ripening in tomato

**Figure 2:**
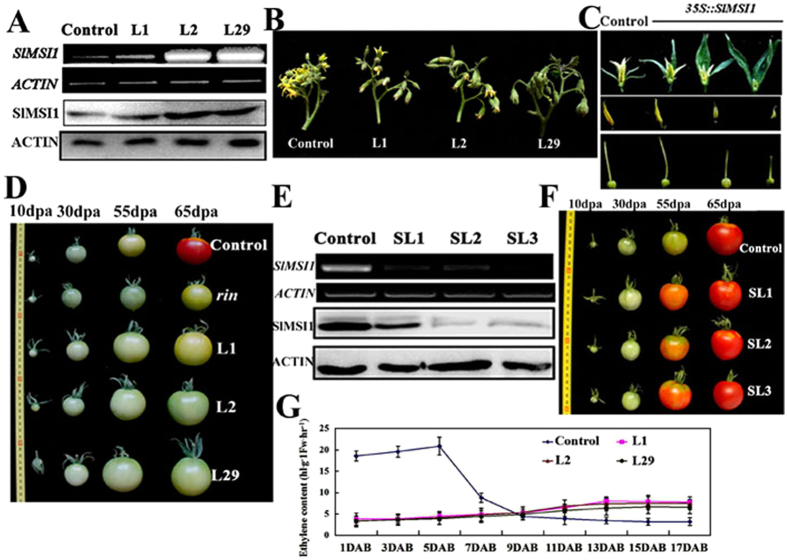
SlMSI1 affects fruit ripening in tomato. (**A**) SlMSI1 transcript levels and protein abundance in the control and 3 overexpression transgenic lines. RNA and protein were extracted from tomato leaves. Overexpression lines L1, L2 and L29 contained *35S::SlMSI1-GFP*. Transgenic plants carrying empty vector (*35S::GFP*) were used as the control. (**B**) Abnormal flowers of overexpression transgenic plants. (**C**) Dissections of abnormal flowers. (**D**) Changes in fruit ripening in the control, mutant *rin* and three overexpression lines at different stages. (**E**) SlMSI1 transcript levels and protein abundance in the control and suppression transgenic lines. RNA and protein were extracted from tomato leaves. Suppression lines SL1, SL2 and SL3 contained a 35S-driven *SlMSI1* antisense cDNA fragment. SlMSI1 RNA and protein samples were derived from the same experiment, and electrophoretic gels and protein blots were processed in parallel. (**F**) Ripening comparison among the control and *SlMSI1* suppression lines. (**G**) Production of ethylene in the control and *SlMSI1* overexpression lines. Fresh fruits from different days after the breaker stage were sealed in airtight vials, and 1 ml of gas was sampled from the headspace after 24 h. DAB, days after breaker.

To characterize the function of *SlMSI1 in planta*, transgenic tomatoes containing *pBIN-SlMSI1-GFP* and empty vector *pBIN-GFP* were obtained. Three *SlMSI1-GFP* overexpression lines L1, L2 and L29 were chosen for further investigation, and a *pBIN-GFP* line was used as the control. The overexpression lines generated more transcripts and proteins of SlMSI1 than the control (Fig. 2A). Compared with the control plants, *SlMSI1* overexpression transgenic lines displayed abnormal flowers with larger sepals and transgenic line L29 showed the indeterminacy inflorescences (Fig. 2B). Additionally, floral dissection revealed smaller stamens and pistils in the overexpression transgenic plants (Fig. 2C). Interestingly, all transgenic tomato fruits produced non-ripening fruits, even at the mature stage, which were highly similar to those in *rin* mutant tomato^30^ (Fig. 2D). Additionally, the overexpression transgenic fruits displayed increased pericarp firmness during the mature green, breaker and mature stages (Supplementary Fig. 1). These results suggest that *SlMSI1* might play a novel role in fleshy fruit development.

Furthermore, a specific antisense *SlMSI1* cDNA fragment was used to construct a suppression vector, which was then genetically transformed into tomato. Finally, 3 suppression lines, SL1, SL2, and SL3, were selected from among the 17 transgenic suppression lines for further investigation. The three lines showed markedly decreased SlMSI1 transcript and protein levels (Fig. 2E). As a result, the 3 suppression lines generated fruits that ripened earlier than the control fruits (Fig. 2F). Additionally, no other obvious changes were found during fruit development and post-harvest. Taken together, these finding suggest that *SlMSI1* inhibits fruit ripening in tomato.

During fruit ripening, the tomato releases a high quantity of ethylene gas that ripens its fruits. We hypothesized that the *SlMSI1* overexpression transgenic lines, which displayed non-ripening fruits, failed to produce ethylene (Fig. 2D). To verify our assumption, we measured ethylene production in the control and transgenic fruits at the breaker stage. The control fruits exhibited a rapid and strong increase in ethylene production; the ethylene level increased to a maximum value at 5 days after the breaker stage and then decreased suddenly until fruit ripening (Fig. 2F). In contrast, there were no obvious changes in the ethylene level in *SlMSI1* overexpression transgenic tomato fruits during ripening (Fig. 2G). Furthermore, the overexpression transgenic tomato fruits failed to ripen when they were treated with exogenous ethylene. These results indicate that the climacteric of ethylene was absent in *SlMSI1* overexpression transgenic fruits during ripening.

### SlMSI1 inhibits fruit ripening by repressing the expression of *RIN* and its regulon genes

**Figure 3:**
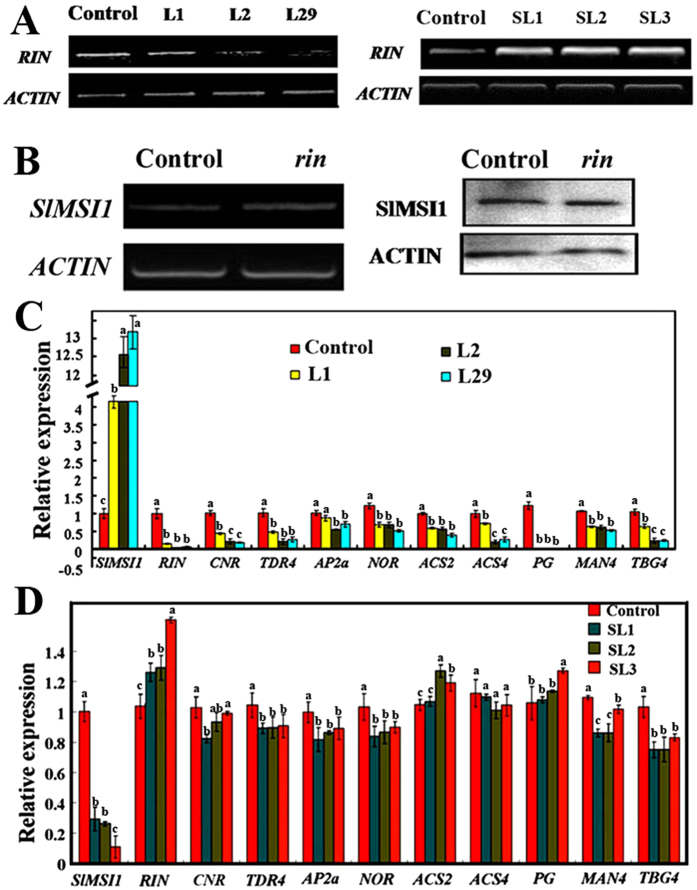
Expression profiles in *rin* mutant, control and transgenic tomatoes. (**A**) Expression of *RIN* in control, *SlMSI1* overexpression and suppression transgenic tomato plants. RNAs were extracted from breaker fruits. (**B**) Transcript and protein levels of SlMSI1 in the *rin* mutant. SlMSI1 RNA and protein samples were derived from the same experiment, and electrophoretic gels and protein blots were processed in parallel. (**C,D**) qRT-PCR expression analysis of the *RIN* and ripening-related, ethylene-related and cell wall modification genes in *SlMSI1* overexpression (**C**) and suppression (**D**) transgenic breaker fruits. The same letter in the same growing season means no significant differences among three biological replicates (P < 0.05). Error bars represent SE.

*SlMSI1* overexpression transgenic tomatoes displayed enlarged sepals and non-ripening fruits that were highly similar to, and even more serious than, those of the *rin* mutant (Fig. 2D). It was assumed that *SlMSI1* might inhibit ripening via repressed *RIN* expression. To test our hypothesis, semi-quantitative PCR was first performed using control and transgenic tomato fruits. This expression analysis demonstrated that *RIN* transcripts were markedly decreased in the three overexpression lines compared with the control (Fig. 3A). Additionally, no changes in the *SlMSI1* transcript and protein levels were found in the *rin* mutant (Fig. 3B), suggesting that *SlMSI1* might act upstream of *RIN* in regulating fruit ripening.

In tomato, RIN appears to be a master regulator that is critical for the transcriptional regulation of ripening initiation and progression^21^,^22^,^23^. Correspondingly, the transcript levels of genes downstream of *RIN*, such as ripening-related, ethylene-related and cell-wall modification genes, were also examined. The results showed that the ripening-related genes *CORLORLESS NON-RIPENING (CNR*), *TDR4, APETALA2 (AP2a*), and *NON-RIPENING (NOR*) were decreased to different degrees (Fig. 3C). Furthermore, the expression levels of two ethylene biosynthetic genes, *1-aminocyclopropane-1-carboxylic acid (ACC) synthase 2 (ACS2*) and *ACS4*, were also reduced in overexpression transgenic tomatoes (Fig. 3C). Certain cell-wall modification genes showed a slight decrease, including *Polygalacturonase (PG*), *Endo-(1,4)- β-mannanase 4 (MAN4*), and *β-Galactosidase 4 (TBG4*), which are thought to be related to the softening and shelf life of fruit (Fig. 3C).

Finally, qRT-PCRs were performed to detect the above genes transcription levels in *SlMSI1* suppression transgenic lines. In accordance with our hypothesis, the transcript levels of *RIN* and its regulons (ripening-related, ethylene-related and cell-wall modification genes) showed little change in the suppression lines (Fig. 3D). Taken together, these results suggest that *SlMSI1* inhibits fruit ripening by repressing the expression of *RIN* and its downstream genes in tomato.

### SlMSI1 suppresses a large set of fruit-ripening genes, including, but not limited to, *RIN* and its regulon

**Figure 4:**
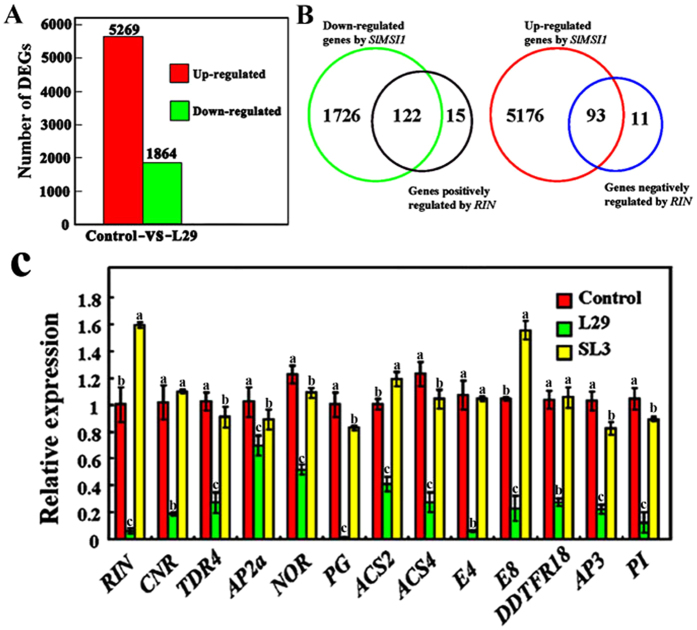
SlMSI1 target genes identified using RNA-seq. (**A**) The numbers of genes that were up-regulated and down-regulated in the overexpression line L29 compared with the control. (**B**) The overlapping gene sets are controlled by *SlMSI1* and *RIN*. The larger circles indicate the potential target genes of *SlMSI1*, and the smaller circles show the potential genes that are positively or negatively regulated by *RIN* as reported by Fujisawa *et al*.^19^. (**C**) qRT-PCR analysis of *SlMSI1* target genes in fruits at the breaker stage of L29 and the control. The same letter in the same growing season means no significant differences among three biological replicates (P < 0.05). Error bars represent SE.

*SlMSI1* overexpression transgenic fruits exhibited a more distinct non-ripening phenotype than the *rin* fruits, suggesting that *SlMSI1* likely inhibits fruit ripening by regulating other genes in addition to *RIN* and its regulon. To test this hypothesis, RNA-seq assays were conducted using *SlMSI1* overexpression transgenic line L29 and control fruits at the mature green stage. The results showed that 5269 genes were up-regulated and 1864 genes were down-regulated in overexpression line L29 by more than two-fold compared to the control (Fig. 4A). The down-regulated genes included *RIN* and most genes in its regulon (Fig. 4B, Supplementary Table S1). Additionally, the expression levels of other ripening genes, such as *E4* and *E8*, were repressed by *SlMSI1* (Fig. 4C), which explains why the non-ripening phenotype of *SlMSI1* transgenic fruits is more pronounced than that of the *rin* mutant. Therefore, these results indicate that *SlMSI1* controls fruit ripening genes that include, but are not limited to, *RIN* and its regulon during fruit ripening. In addition, *SlMSI1* regulated the expression levels of homeotic genes, i.e., *AP3* and *PI*, and its transgenic lines exhibited abnormalities in the floral and other reproductive phenotypes (Fig. 4C).

### Genetic manipulation of *SlMSI1* and *RIN* prolongs fruit shelf life

**Figure 5:**
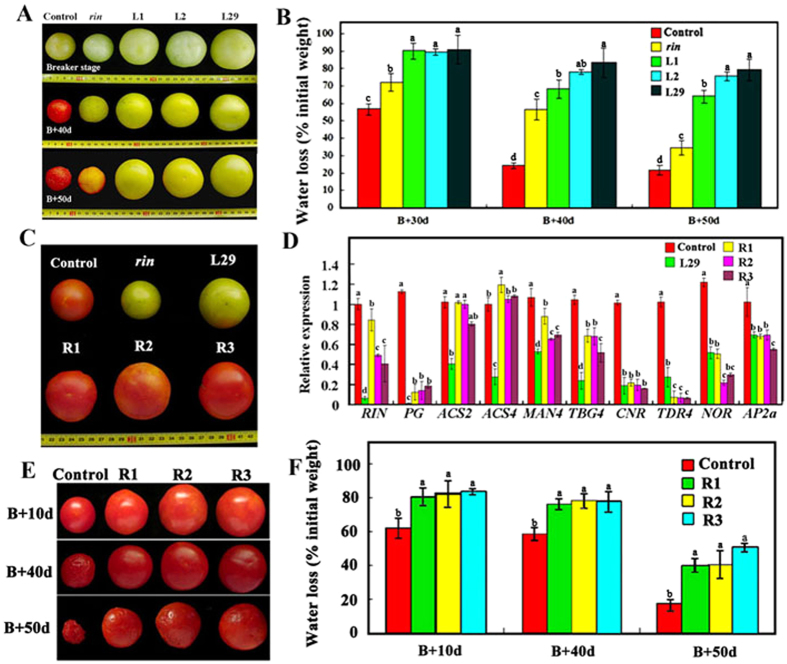
Genetic manipulation of *SlMSI1* and *RIN* prolongs fruit shelf life. (**A**) Ripening comparison among the control, mutant *rin*, and *SlMSI1* overexpression lines L1, L2 and L29. (**B**) The dehydration statistics in the transgenic and control fruits at 30, 40, and 50 days after the breaker stage. The fresh weight of the fruit at the breaker stage was recorded as the starting point. The water loss was calculated as the percentage difference between the starting weight and each subsequent measurement. (**C**) Ripening comparison among the control, *rin* mutant, *SlMSI1* overexpression line L29 and recovery lines (R1, R2 and R3). The background for the recovery lines is L29. (**D**) The relative expression of the ripening genes in lines L29, R1, R2, R3 and the control. The same letter in the same growing season means no significant differences among three biological replicates (P < 0.05). Error bars represent SE. (**E**) Comparison of the fruit shelf life between the control and the recovery lines. (**F**) The dehydration statistic for the fruits in the control and three recovery lines. (**B**), breaker. Control in (**A–F**) indicates transgenic tomatoes carrying empty vector *pBIN-GFP*.

To examine the shelf life, breaker fruits of the control, *rin* mutant and 3 overexpression lines (L1, L2 and L29) were harvested and placed at room temperature. The control fruit turned completely red 40 days after harvest (DAH) and began to shrink, whereas the *rin* fruits exhibited a pale yellow color and started to shrink at 50 DAH. In contrast, the fruits of the 3 overexpression lines remained green at 50 DAH (Fig. 5A). In addition, dehydration assays were performed using the fruits at 30, 40 and 50 days after the breaker stage (B + 30 d, B + 40 d and B + 50 d, respectively). The results showed that there was less water loss in the 3 overexpression lines than in the control and even less water loss in the *rin* fruits at all 3 tested stages (Fig. 5B).

Furthermore, *RIN (Solyc05g012020.2.1*) was genetically transformed into *SlMSI1* transgenic line L29 to produce three recovery lines, R1, R2 and R3, with a L29 background to investigate expression rescue (Fig. 5C). The three lines produced many more *RIN* transcripts than L29 but fewer than the control. Additionally, the expression levels of the genes in the *RIN* regulon, such as *PG, ACS2, ACS4, MAN4* and *TBG4,* were partially recovered in the 3 recovery lines (Fig. 5D). However, the transcript levels of the other ripening genes, such as *CNR, NOR, TDR4*, and *AP2a*, did not increase in the three recovery lines compared with L29. As a result, the fruits of the R1, R2 and R3 lines ripened more quickly than did the L29 fruits. Compared with the control fruits, the fruits of the three recovery lines exhibited a noticeably prolonged shelf life, as indicated by their non-shrunken appearance and reduced dehydration (Fig. 5E,F).

## Discussion

MSI1 protein contains WD-40 repeat domains and is a member of the evolutionarily conserved PcG complexes^11^. In *Arabidopsis*, MSI1 has been found to interact with other PcG proteins to form diverse PRC2 complexes, i.e., the VRN complex that accelerates flowering in response to prolonged exposure to cold^12^,^13^; the EMF complex that controls vegetative development and the transition to flowering^9^,^14^; and the FIS complex that specially functions in female gametophyte and endosperm development^10^,^11^. In addition to the PRC2 complex, MSI1 is an essential component of the *Arabidopsis* chromatin assembly factor (CAF-1) complex, as are the proteins FASCIATA 1 (FAS1) and FASCIATA 2 (FAS2)^31^. The CAF-1 complex is conserved in yeast, *Drosophila* and mammals and is essential for the deposition of the heterodimer H3-H4 at the replication fork^32^,^33^. In tomato, LeMSI1/SlMSI1 has been found to interact with distinct proteins during fruit development^28^, suggesting that SlMSI1 might be involved in regulating fruit development. Here, we found that *SlMSI1* overexpressing lines displayed the abnormal flowers with larger sepals and indeterminacy inflorescences (Fig. 2B,C). *MACROCALYX (MC*) affects inflorescence determinacy and sepal development. qRT-PCR data showed that *MC* was decreased in all three ovexpression transgenic lines and recover lines, but changed a little in suppression lines (Supplementary Fig. 2). The results suggested that abnormal flower development might be induced by *MC*. Additionally, overexpressed *SlMSI1* resulted in non-ripening fruits with a long shelf life. In *Arabidopsis*, MSI1 has been shown to function in flower and seed development^34^,^35^. We found that *SlMSI1* overexpression and suppression transgenic tomatoes were affected with respect to both flower development and fruit ripening (Fig. 2E), which is indicative of a novel role for *SlMSI1* in fleshy fruit species.

Fruit ripening is a complex process accompanied by numerous developmental and metabolic changes^36^,^37^. In this study, *SlMSI1* inhibited fruit ripening by negatively regulating ripening-related genes (*RIN, CNR* and *TDR4*), ethylene synthesis genes (*ACS2* and *ACS4*) and cell-wall modified genes (*PG* and *MAN4*), all of which have been reported to be regulated by *RIN* 19,22. Previous studies have noted the complicated regulatory network of *RIN* and its target genes during fruit ripening; however, the potential role and mechanism of how these regulators act upstream of *RIN* to control fruit ripening remain unknown. Here, it was found that *RIN* and its target genes were markedly down-regulated in *SlMSI1* overexpression transgenic tomatoes. Furthermore SlMSI1 protein and transcript levels did not change in the *rin* mutant, indicating that *SlMSI1* acts upstream of *RIN* and regulates fruit ripening as a negative modulator. Therefore, our findings provide a new component of the emerging knowledge of mechanisms regulating fleshy fruit ripening.

Recently, it has been reported that DNA methylation in promoters of typical ripening genes are gradually decreased during ripening^16^,^17^, providing insight into the role of epigenetic DNA modifications on fruit ripening and their potential utilization in breeding programs for improving fruit shelf life. Furthermore, another PcG protein, SlEZ2, regulates vegetative and reproductive development of tomato fruits and has a strong influence on the global level of H3K27me3^27^. Notably, SlMSI1 belongs to a subfamily of WD-40 repeat proteins, which are evolutionarily conserved developmental suppressors that act via DNA methylation and histone methylation^1^,^3^. In this study, it was found that the protein and transcript levels of SlMSI1 were clearly decreased at the onset of ripening during fruit development, whereas the expression levels of *RIN* and its target genes changed in an inverse manner. It appears that the SlMSI1-associated PRC2 complex and the related DNA methylation modification likely contributed to the inhibition of the *RIN* regulon and fruit ripening. Recently, numerous transcription factors associated with H3K27me3 markers have been identified, such as certain MADS-box factors^38^.

One major goal of fruit improvement is to reduce post-harvest wastage. A long shelf life is a desired trait for both breeders and purchasers. With an increasing understanding of the molecular mechanisms by which plants regulate fruit ripening and the advent of genetic engineering technologies, researchers have focused on finding new strategies to address problems related to fruit shelf life. Commercial breeding is currently focused on genetic polymorphisms. In this study, it was found that the PcG protein SlMSI1 negatively regulates *RIN* and other ripening genes, and genetic manipulation of *SlMSI1* and *RIN* successfully prolonged the fruit shelf life, demonstrating that SlMSI1 is a novel regulator suitable for use in genetic engineering modification of fruit shelf life.

## Materials and Methods

### Plant materials and growth conditions

Tomato cultivars (*Solanum lycopersicum* cv. Ailsa Craig) and *rin* mutant were grown in greenhouses under natural conditions (16 h supplemental lighting at 25 °C and 8 h at 16 °C). Fruit ripening stages of tomato were divided according to days after anthesis (dpa) and color changes^39^. Flowers were tagged at anthesis. In wild type fruits, mature green (MG) were defined as 45 dpa and were characterized as fully expanded unripe fruit with mature seeds. Breaker (B) fruits were defined as 55 dpa and the color change from green to yellow. Fruits at 65 DAF were fully ripe and can vary substantially among cultivars.

### RNA extract, qRT-PCR and RNA-seq analysis

Harvested tomato tissues were immediately frozen in liquid nitrogen and stored at −80 °C. Total RNAs were isolated using Trizol (Invitrogen) according to the manufacturer’s instructions and used for PCR and RNA-seq analysis. For qRT-PCR analysis, the first-strand cDNA was synthesized using an M-MLV system. The qRT-PCR was performed using specific primers (Supplementary Table S2) as described by Liu *et al*.^40^. Three technical replicates were performed for each sample every time and three biological repeats were performed. The relative quantitative values were calculated using the 2^−△△Ct^ method^41^. The specificity of the amplification was determined by performing a dissociation curve analysis.

For RNA-seq, total RNA samples were prepared from the fruits at the breaker stage (55 dpa) using Trizol (Invitrogen) and purified with the RNeasy Plus Kit (Qiagen). Then, the RNA was analyzed using a high-throughput parallel sequencing using an Illumina genome analyzer II according to the manufacturer’s instructions. The false discovery rate (FDR) was set at 1% to determine the threshold of the P-value in multiple tests and analyses by manipulating the FDR value. P < 0.001 and the absolute value of log2 ratio >1 were used as the threshold to judge the significance of the gene expression difference according to Audic and Claverie^42^.

### Protein extraction and Western blot analysis

SlMSI1 protein level was detected using tomato leaves and breaker stage fruits, respectively. All proteins were extracted as described by Li *et al*.^43^. The protein concentration was determined using the Bradford reagent (Sigma-Aldrich) with bovinese rumalbum as a standard.

Anti-SlMSI1 polyclonal antibody was commissioned from GenScript Company. Protein extracts (30 μL) were lysed in gel-loading buffer containing 50 mM Tris-HCl (pH 6.8), 100 mM dithiothreitol (DTT), 2% sodium dodecyl sulfate (SDS), 0.1% bromophenol blue, and 10% glycerol. Fifty micrograms of protein was resolved using SDS-polyacrylamide gel electrophoresis (PAGE) and electrically blotted onto a nylon filter (Roche). The filters were blocked with phosphate-buffered saline (PBS) containing 15% nonfat milks and incubated with specific antibody of anti-SlMSI1 (1:5000), polyclonal antibody ACTIN (1:5000, Abcam) was used as control, respectively. After washing with PBS containing 0.5% Tween 20, the bound primary antibody was detected using anti-mouse IgG or anti-rabbit IgG (1:10000, Abcam). After washing, the anti-specific antibodies or anti-control antibodies bound proteins were visualized using Immobilon Western chemiluminescent HRP substrate kit (Millipore).

### Generation of transgenic tomato plants

Overexpression constructs were made by cloning the *SlMSI1 (Solyc01g104510.2.1*) cDNA using the *Bam*HI restriction enzyme into the pBIN-GFP vector. The *SlMSI1* suppression construct was generated using antisense sequence of its 3′-UTR. All constructs were sequence confirmed and transformed into *Agrobacterium tumefaciens* (strain LBA4404) via electroporation. Tomato transformation was performed as described by Ellul *et al*.^44^. The transgenic plants were verified with PCR using two sets of primers. 35S and gene-specific reverse primers were used to amplify the region encompassing the end of the 35S promoter and transgene, while NPT2F and NPT2R were used to amplify the kanamycin resistance gene.

*RIN* was genetically transformed into *SlMSI1* transgenic line L29. The recovery constructs were made by inserting the *RIN* cDNA (*Solyc05g012020.2.1*) using *Xcm*1 restriction enzyme into the pCXSN vector^45^. The resultant constructs were genetically transformed into *SlMSI1* overexpression transgenic line L29 with an *Agrobacterium* method. The transgenic plants were chosen by both kanamycin- and hygromycin- resistance. Gene-specific primers were used to amplify the *RIN* gene. The specific primers are listed in Supplementary Table S2. Homozygous transgenic lines were used for investigation.

### Dehydration assay

Dehydration analysis was performed using 10 fruits that were harvested at the breaker stage. The fruits were kept at room temperature for 30 days, 40 days and 50 days. The fresh weights were recorded every 10 days. The fresh weight of the fruit at the breaker stage was recorded as the starting point. The water loss was calculated as the percentage difference in weight between the starting weight and each subsequent measurement.

### Ethylene measurement

Fruits of breaker, 1 day after breaker (B + 1), B + 3, 5, 7, 9, 11, 13, 15, and 17 were harvested and placed in open 500 ml jars for 2 h to minimize the effect of wound ethylene caused by picking. Jars were then sealed and incubated at room temperate for 24 h, 1 ml of headspace gas was injected into a Hewlett-Packard 5890 series gas chromatograph equipped with a flame ionization detector (FID). Samples were compared with reagent grade ethylene standards of known concentration and normalized for fruit weight^46^.

### Firmness measurement

Firmness measurements were made by fruit firmness tester (GY-2, China). The maximum force recorded at 10 mm of compression was used as an estimation of the fruit firmness from the averaged value of at least three tested fruits with a minimum of three compressions per fruit.

### Statistical analysis

The mean values of qRT-PCR and dehydration measurement were taken from the measurements of three biological replicates, and SE was calculated. Statistical analysis of the data was tested with ANOVA and significant difference at the 5% level using the SPSS 16.0 software package.

## Additional Information

**How to cite this article**: Liu, D.-D. *et al*. Polycomb-group protein SlMSI1 represses the expression of fruit-ripening genes to prolong shelf life in tomato. *Sci. Rep.* **6**, 31806; doi: 10.1038/srep31806 (2016).

## Supplementary Material

Supplementary Information
