# Supplementary material for: Polycomb-group protein SlMSI1 represses the expression of fruit-ripening genes to prolong shelf life in tomato
Source: Sci Rep. 2016 Aug 25;6:31806. doi: 10.1038/srep31806 (PMC4997261; doi:10.1038/srep31806)
Supplement: Supplementary Information [file srep31806-s1.pdf]

Polycomb-group protein SlMSI1 represses the expression of fruit-ripening genes to prolong shelf life in tomato

Dan-Dan Liu[a,b,c], Li-Jie Zhou[a,b], Mou-Jing Fang[a,b], Qing-Long Dong[a,b], Xiu-Hong An[a,b], Chun-Xiang You[a,b], and Yu-Jin Hao[a,b,*]

[a]National Key Laboratory of Crop Biology; [b]College of Horticulture Science and Engineering, Shandong Agricultural University, Tai-An, Shandong 271018, China. [c]College of Agriculture, Yunnan University, Kunming, Yunnan 650091, China.[*]Correspondence to haoyujin@sdau.edu.cn.

Supplementary information list:

Supplementary Figure 1: Pericarp firmness as measured by compression in mature green (MG), breaker (BR) and mature (MR) fruits.

Supplementary Figure 2. Expression of *MACROCALYX* (*MC*) in *SlMSI1* overexpression, suppression and recover transgenic tomato plants.

Supplementary Table 1: Common genes regulated by *SlMSI1* and *RIN* in fruits.

Supplementary Table 2: Oligonucleotide primers used in this study.

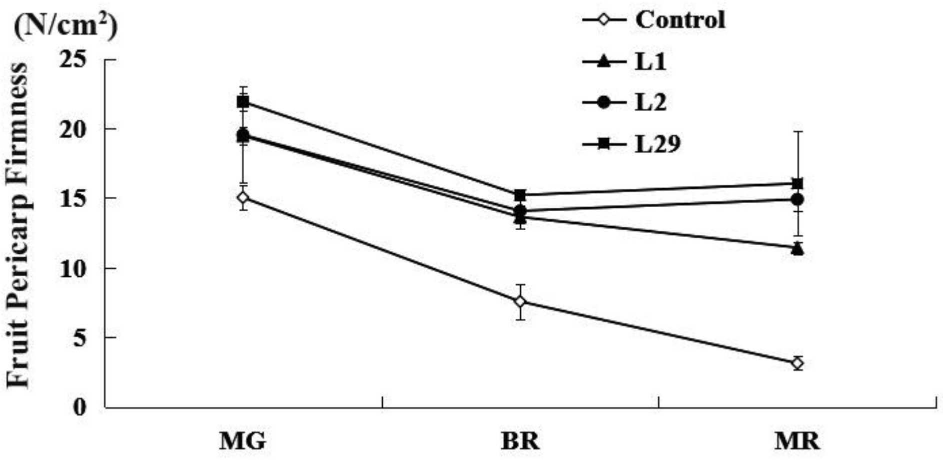

Supplementary figure 1. Pericarp firmness as measured by compression in mature green (MG), breaker (BR) and mature (MR) fruits.

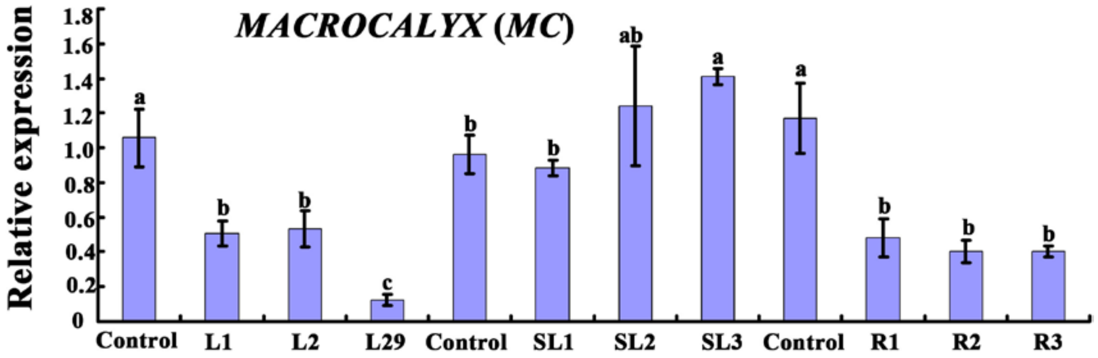

Supplementary figure 2. Expression of *MACROCALYX* (*MC*) in *SlMSI1* overexpression, suppression and recover transgenic tomato plants. RNAs were extracted from breaker fruits. L1, L2 and L29 represented *SlMSI1* overexpression lines; SL1, SL2 and SL3 represented suppression lines; R1, R2 and R3 represented recovery lines. Significant differences ($P < 0.05$) are denoted by different letters. Error bars represent SE.

Supplementary Table 1. Common genes regulated by *SlMSI1* and *RIN* in fruits. Red and green color indicate the up-regulated and down-regulated genes by *SlMSI1*, repestively.

Gene ID	log2 Ratio(L29/Control)	Up-Down-Regulation (L29/Control)	P-value	Annoation
Solyc02g087970.1.1	10.33066067	Up	0	mini zinc finger protein
Solyc02g071520.2.1	9.383870571	Up	7.58E-09	bidirectional sugar transporter SWEET2a-like
Solyc04g005800.2.1	5.300195386	Up	5.47E-45	homeobox-leucine zipper protein HAT5-like
Solyc02g090680.2.1	4.686804476	Up	4.04E-28	kip-related protein [Solanum lycopersicum var. cerasiforme]
Solyc07g042390.1.1	4.611292032	Up	9.43E-153	PREDICTED: 21 kDa protein-like
Solyc04g007500.1.1	4.593948376	Up	0	PREDICTED: E3 ubiquitin-protein ligase RHA2A-like [Vitis vinifera]
Solyc01g091770.2.1	4.307215813	Up	7.00E-21	RING-H2 finger protein ATL16-like
Solyc03g083100.2.1	4.215661875	Up	0	protein IQ-DOMAIN 1-like
Solyc07g062550.2.1	3.918395835	Up	2.27E-22	UDP-N-acetylglucosamine 1-carboxyvinyltransferase 2-like
Solyc08g048290.2.1	3.760073571	Up	0	probable inositol transporter 2-like
Solyc01g007200.2.1	3.697530883	Up	2.14E-24	uncharacterized protein LOC101262337
Solyc02g090620.2.1	3.624630336	Up	2.21E-63	uncharacterized protein LOC101247177
Solyc02g086300.2.1	3.460968689	Up	3.46E-223	protein NtpR-like
Solyc04g009790.2.1	3.457216554	Up	2.41E-114	uncharacterized protein LOC101251972
Solyc01g095630.2.1	3.390460227	Up	0	WRKY transcription factor
Solyc06g062670.2.1	3.059784287	Up	0	zinc finger protein NUTCRACKER-like isoform 1
Solyc08g078300.2.1	3.037669988	Up	1.86E-73	homeobox-leucine zipper protein HAT4-like
Solyc02g062690.2.1	3.017605439	Up	5.88E-24	transcription factor bHLH63-like
Solyc01g104850.2.1	3.017605439	Up	1.75E-91	serine carboxypeptidase-like 42-like
Solyc01g104030.2.1	2.982618532	Up	0	potassium channel AKT2/3-like
Solyc08g067410.1.1	2.902413536	Up	8.05E-55	3-ketoacyl-CoA synthase 11-like
Solyc01g099290.2.1	2.800624376	Up	7.88E-40	cullin-1-like isoform 1
Solyc06g068520.2.1	2.772234172	Up	2.72E-174	hydroxyproline-rich systemin precursor
Solyc02g093150.2.1	2.62724497	Up	6.82E-247	AP2 transcription factor SlAP2c
Solyc06g075370.2.1	2.592518394	Up	3.48E-149	dof zinc finger protein DOF2.1-like
Solyc10g084990.1.1	2.589282778	Up	4.38E-29	phosphatidylinositol:ceramide inositolphosphotransferase 1-like
Solyc02g078150.2.1	2.522425643	Up	3.36E-165	uncharacterized protein LOC101251740
Solyc12g088750.1.1	2.482836012	Up	2.28E-82	probable serine/threonine-protein kinase At1g01540-like
Solyc02g078240.2.1	2.452115682	Up	3.58E-29	phosphoserine phosphatase 1-like
Solyc10g084340.1.1	2.428070208	Up	5.09E-06	thylene-responsive transcription factor RAP2-7-like
Solyc02g068340.2.1	2.402783766	Up	1.39E-171	kinesin-like calmodulin-binding protein-like
Solyc06g076040.2.1	2.389650325	Up	0	U-box domain-containing protein 13-like
Solyc07g062530.2.1	2.386223131	Up	0	phosphoenolpyruvate carboxylase-like
Solyc02g064960.2.1	2.351108226	Up	2.57E-09	AP2 transcription factor SlAP2b

Solyc04g081290.2.1	2.214083025	Up	0	Cytokinin riboside 5'-monophosphate phosphoribohydrolase LOG1-like
Solyc06g083130.2.1	2.205677787	Up	7.01E-05	dCTP pyrophosphatase 1-like
Solyc07g017540.2.1	2.19495138	Up	2.19E-28	DNA repair protein RAD51 homolog
Solyc07g008110.2.1	2.126581005	Up	2.45E-221	dicyanin precursor
Solyc01g088100.2.1	2.122531074	Up	0	zinc finger CCCH domain-containing protein 53-like isoform 2
Solyc09g059430.2.1	2.094384142	Up	2.77E-174	RPM1-interacting protein 4-like
Solyc07g018340.2.1	2.05982692	Up	9.76E-67	DNA mismatch repair protein MSH7
Solyc07g006890.1.1	2.038255292	Up	0	cytochrome P450 94A1-like
Solyc03g120450.2.1	2.030393869	Up	1.75E-58	aspartate aminotransferase-like
Solyc11g008140.1.1	1.999226909	Up	1.08E-10	probable pectate lyase 12-like
Solyc03g112060.2.1	1.995903273	Up	1.80E-55	uncharacterized protein LOC101246910
Solyc06g083230.2.1	1.94073126	Up	4.20E-66	GTP cyclohydrolase I
Solyc05g009820.2.1	1.893435857	Up	2.02E-68	probable galacturonosyltransferase-like 3-like
Solyc10g080560.1.1	1.877055039	Up	1.88E-08	DNA-3-methyladenine glycosylase-like
Solyc03g117810.2.1	1.869628584	Up	3.30E-61	ABC transporter I family member 17-like
Solyc02g078590.1.1	1.853376043	Up	3.16E-43	uncharacterized protein At4g19900-like
Solyc04g054980.2.1	1.836831389	Up	0	lipoxygenase homology domain-containing protein 1-like
Solyc05g013570.2.1	1.800624376	Up	4.94E-24	BTB/POZ domain-containing protein At1g67900-like
Solyc11g072600.1.1	1.784214018	Up	1.11E-18	AP2 transcription factor SlAP2d
Solyc02g085340.1.1	1.781038288	Up	2.68E-47	scarecrow-like protein 4-like
Solyc12g096500.1.1	1.780549394	Up	7.66E-94	zinc finger protein CONSTANS-LIKE 4-like
Solyc02g083990.2.1	1.744097702	Up	1.82E-123	uncharacterized protein LOC101257129
Solyc08g077060.2.1	1.741228093	Up	4.68E-44	protein LOL1-like isoform 3
Solyc01g109930.2.1	1.731561055	Up	4.93E-70	bifunctional phosphatase IMPL2, chloroplastic-like
Solyc03g114730.2.1	1.723258971	Up	3.44E-171	uncharacterized protein At1g04910-like
Solyc04g053120.2.1	1.64258344	Up	2.36E-173	4-alpha-glucanotransferase, chloroplastic/amyloplastic-like
Solyc03g117300.2.1	1.625273568	Up	7.14E-23	uncharacterized protein LOC101263299
Solyc01g111400.2.1	1.571805686	Up	2.19E-142	subtilisin-like protease-like
Solyc09g010630.2.1	1.566462924	Up	0	heat shock cognate 70 kDa protein 2-like
Solyc06g083660.2.1	1.554587037	Up	5.88E-244	uncharacterized protein LOC101266733
Solyc02g089630.2.1	1.516146515	Up	0	proline dehydrogenase 2, mitochondrial-like
Solyc02g090510.2.1	1.504522269	Up	8.37E-11	CDPK-related kinase 5-like
Solyc05g005700.2.1	1.49523951	Up	0	aldehyde dehydrogenase family 2 member B7, mitochondrial-like
Solyc04g082480.2.1	1.477953581	Up	1.98E-58	protein NLP4-like
Solyc02g083880.2.1	1.446490147	Up	1.30E-39	snakin-2-like
Solyc10g079130.1.1	1.439410608	Up	0	calcium-dependent protein kinase 32-like
Solyc01g102310.2.1	1.439383357	Up	5.72E-296	uncharacterized protein LOC101267652
Solyc05g009550.2.1	1.400506498	Up	0	uncharacterized protein LOC101258366
Solyc02g085030.2.1	1.370015737	Up	2.32E-134	uncharacterized protein LOC101259403

Solyc02g064680.2.1	1.302974988	Up	0	calcium-transporting ATPase 2, plasma membrane-type-like
Solyc07g008100.2.1	1.205677787	Up	2.26E-07	uncharacterized protein LOC101245669
Solyc09g005080.1.1	1.185401739	Up	7.48E-155	verticillium wilt disease resistance protein
Solyc05g051660.1.1	1.162917215	Up	9.45E-41	probable carboxylesterase 13-like
Solyc12g099280.1.1	1.033925432	Up	5.45E-37	uncharacterized protein LOC101259137
Solyc10g050860.1.1	1.014093319	Up	2.94E-178	DNA-binding protein
Solyc10g080210.1.1	-16.85268924	Down	0	polygalacturonase-2 precursor (PG)
Solyc05g012020.2.1	-9.586155432	Down	2.12E-104	MADS-box transcription factor (*RIN*)
Solyc05g050010.2.1	-9.414542039	Down	0	1-aminocyclopropane 1-carboxylate synthase (*ACS4*)
Solyc10g083400.1.1	-7.907062438	Down	0	cytochrome P450 94A1-like
Solyc07g052960.1.1	-7.610188814	Down	0	scarecrow-like protein 32-like
Solyc09g008170.1.1	-7.387313088	Down	0	auxin-induced protein X10A-like
Solyc09g010210.2.1	-7.383091955	Down	0	endo-1,4-beta-glucanase precursor
Solyc01g006540.2.1	-6.556935422	Down	0	lipoxygenase
Solyc01g088590.2.1	-6.351977368	Down	0	putative invertase inhibitor-like
Solyc06g076320.1.1	-5.967463927	Down	0	uncharacterized protein LOC101245955
Solyc07g064410.1.1	-5.917032576	Down	3.70E-166	transmembrane protein 189-like
Solyc12g098710.1.1	-5.87919294	Down	0	15-cis-zeta-carotene isomerase, chloroplastic-like isoform 1
Solyc03g111720.2.1	-5.675279178	Down	0	Full=Fruit-ripening protein E4 (*E4*)
Solyc04g005020.2.1	-5.582224773	Down	0	uncharacterized WD repeat-containing protein alr3466-like
Solyc01g098110.2.1	-5.458525116	Down	0	acyltransferase-like protein At1g54570, chloroplastic-like
Solyc06g051800.2.1	-5.396136181	Down	0	expansin precursor
Solyc02g077710.1.1	-5.186234987	Down	0	uncharacterized protein LOC101261288
Solyc01g095080.2.1	-4.923028303	Down	0	ACC synthase 2 (*ACS2*)
Solyc08g014130.2.1	-4.65402408	Down	0	Alpha-isopropylmalate synthase A
Solyc08g066690.2.1	-4.564694097	Down	0	probable glycosyltransferase At5g03795-like
Solyc04g080250.2.1	-4.551712223	Down	0	deoxyuridine 5'-triphosphate nucleotidohydrolase-like
Solyc08g005770.2.1	-4.504052854	Down	0	alcohol acyl transferase
Solyc03g098290.2.1	-4.207916296	Down	0	sucrose synthase 6-like
Solyc06g074710.1.1	-4.207479979	Down	2.04E-206	agmatine coumaroyltransferase-2-like
Solyc08g079480.2.1	-4.197446391	Down	1.59E-144	uncharacterized protein LOC101265157
Solyc02g070890.2.1	-3.943674723	Down	0	LRR receptor-like serine/threonine-protein kinase FLS2-like
Solyc03g044300.2.1	-3.913507655	Down	0	APETALA2-like protein (*AP2*)
Solyc08g066700.2.1	-3.894319722	Down	0	exosome complex exonuclease RRP46 homolog
Solyc04g064610.2.1	-3.741854793	Down	2.60E-54	bidirectional sugar transporter SWEET1-like
Solyc06g068900.2.1	-3.671259587	Down	0	nudix hydrolase 17, mitochondrial-like
Solyc07g065970.1.1	-3.664529511	Down	0	chaperone protein dnaJ 11, chloroplastic-like
Solyc01g098740.2.1	-3.592766081	Down	1.04E-191	receptor-like serine/threonine-protein kinase ALE2-like
Solyc01g099190.2.1	-3.536476554	Down	0	lipoxygenase
Solyc06g069430.2.1	-3.495979029	Down	0	TDR4 transcription factor (*TDR4*)

Solyc09g089580.2.1	-3.470393777	Down	0	Full=1-aminocyclopropane-1-carboxylate oxidase homolog (*E8*)
Solyc10g079050.1.1	-3.370503828	Down	9.13E-184	transcription factor bHLH130-like
Solyc08g080670.1.1	-3.301282202	Down	4.98E-33	PR5-like protein precursor
Solyc03g115500.2.1	-3.066694484	Down	7.85E-203	heparanase-like protein 1-like
Solyc01g094790.2.1	-2.969554499	Down	0	bifunctional L-3-cyanoalanine synthase/cysteine synthase 2, mitochondrial-like
Solyc01g095900.2.1	-2.912960825	Down	0	clustered mitochondria protein homolog
Solyc07g054060.2.1	-2.879584818	Down	0	uncharacterized protein LOC101263470
Solyc03g005020.2.1	-2.766872886	Down	0	Triacylglycerol lipase 2-like
Solyc10g008470.2.1	-2.764439611	Down	2.24E-120	ELMO domain-containing protein A-like
Solyc10g009430.2.1	-2.716953658	Down	4.06E-215	O-acyltransferase WSD1-like
Solyc10g081650.1.1	-2.652983019	Down	0	CrtISO
Solyc01g008910.2.1	-2.583884796	Down	7.58E-133	scarecrow-like protein 3-like
Solyc03g116630.2.1	-2.578407356	Down	0	uncharacterized protein LOC101253674
Solyc07g047800.2.1	-2.519315661	Down	0	peroxisomal 2,4-dienoyl-CoA reductase SPS19-like isoform 1
Solyc08g081010.2.1	-2.417450174	Down	0	GSH1
Solyc10g085680.1.1	-2.40183903	Down	6.54E-300	serine/threonine-protein kinase STN8, chloroplastic-like
Solyc04g078810.2.1	-2.340756485	Down	0	uncharacterized protein LOC101252176
Solyc12g096570.1.1	-2.307525088	Down	2.97E-134	ARGOS
Solyc09g082460.2.1	-2.300832715	Down	0	homocysteine S-methyltransferase 3-like
Solyc02g085350.2.1	-2.281800739	Down	0	succinate dehydrogenase [ubiquinone] flavoprotein subunit 1, mitochondrial-like isoform 1
Solyc08g078460.2.1	-2.275095864	Down	6.30E-231	inositol 2-dehydrogenase/D-chiro-inositol 3-dehydrogenase-like
Solyc01g081610.2.1	-2.256662844	Down	0	beta-hexosaminidase 1 precursor
Solyc03g117870.2.1	-2.212199664	Down	0	4-coumarate--CoA ligase 1-like
Solyc03g025970.2.1	-2.207757306	Down	0	uncharacterized protein LOC101246025
Solyc03g114360.2.1	-2.18577848	Down	0	probable inactive poly [ADP-ribose] polymerase SRO5-like
Solyc12g010320.1.1	-2.15331893	Down	0	temperature-induced lipocalin
Solyc08g081890.2.1	-2.151417163	Down	5.37E-249	ABC transporter C family member 10-like
Solyc02g083380.2.1	-2.108859977	Down	3.50E-180	uncharacterized protein LOC101245418
Solyc02g036350.2.1	-2.030635891	Down	0	1-aminocyclopropane-1-carboxylate oxidase
Solyc08g061270.1.1	-2.01272267	Down	3.84E-199	uncharacterized protein LOC101264141 isoform 1
Solyc12g005860.1.1	-2.008513555	Down	0	aconitate hydratase, cytoplasmic-like isoform 2
Solyc03g080020.2.1	-1.932042863	Down	2.06E-53	UNC93-like protein 1-like
Solyc10g085880.1.1	-1.925550784	Down	0	UDP-glycosyltransferase 73C4-like isoform 2
Solyc10g006880.2.1	-1.922344333	Down	0	NAC domain protein (*NOR*)
Solyc08g063040.2.1	-1.906599556	Down	0	zinc finger protein NUTCRACKER-like
Solyc01g097880.2.1	-1.90208803	Down	0	guanine deaminase-like isoform 1
Solyc06g072650.1.1	-1.816148576	Down	1.74E-13	auxin-induced protein 10A5-like
Solyc01g009010.2.1	-1.795132489	Down	0	4-diphosphocytidyl-2-C-methyl-D-erythritol kinase, chloroplastic/chromoplastic

Solyc12g099810.1.1	-1.702335092	Down	1.47E-64	crt homolog 3-like
Solyc02g077240.2.1	-1.686171646	Down	0	pyruvate decarboxylase isozyme 1-like
Solyc01g100460.2.1	-1.642777919	Down	4.13E-60	naerobic basic leucine zipper protein
Solyc06g009380.2.1	-1.641429234	Down	0	uncharacterized protein LOC101264013
Solyc07g064420.2.1	-1.596382836	Down	1.23E-122	uncharacterized protein LOC101251905
Solyc10g085280.1.1	-1.560222708	Down	2.51E-24	UDP-glycosyltransferase 76E11-like
Solyc04g071150.2.1	-1.549186987	Down	0	abscisic acid 8'-hydroxylase 3-like
Solyc08g077780.2.1	-1.543671532	Down	2.84E-276	serine/threonine-protein kinase SAPK3-like
Solyc12g099340.1.1	-1.528510723	Down	2.43E-275	calmodulin-binding transcription factor SR3L
Solyc03g083090.2.1	-1.518338076	Down	0	soluble starch synthase 1, chloroplastic/amyloplastic-like
Solyc04g074850.2.1	-1.512389955	Down	0	ripening regulated protein (*DDTFR18*)
Solyc04g081900.2.1	-1.427603656	Down	0	probable nucleoredoxin 2-like
Solyc02g077920.2.1	-1.409055851	Down	0	squamosa promoter binding-like protein (*CNR*)
Solyc07g056390.2.1	-1.367903627	Down	1.58E-170	endoplasmic oxidoreductin-1-like
Solyc08g062820.2.1	-1.356225698	Down	4.44E-131	cytokinin riboside 5'-monophosphate phosphoribohydrolase LOG8-like
Solyc01g096660.2.1	-1.351188907	Down	0	uncharacterized protein LOC543930
Solyc09g061280.2.1	-1.28617531	Down	4.33E-15	KIP1-related-protein 2
Solyc09g065520.2.1	-1.278455723	Down	2.52E-199	selenocysteine methyltransferase-like
Solyc04g011520.2.1	-1.201383266	Down	1.53E-169	protein kinase APK1A, chloroplastic-like
Solyc02g078400.2.1	-1.182101947	Down	0	probable allantoinase 1-like isoform 1
Solyc01g095580.2.1	-1.123025229	Down	1.02E-173	probable indole-3-acetic acid-amido synthetase GH3.5-like
Solyc05g050970.2.1	-1.114987002	Down	1.04E-233	transketolase, chloroplastic-like
Solyc11g065070.1.1	-1.086045378	Down	4.91E-77	hydroxymethylglutaryl-CoA lyase, mitochondrial-like
Solyc09g082470.2.1	-1.085695534	Down	6.49E-80	serine/threonine-protein kinase HT1-like
Solyc09g074270.2.1	-1.06044537	Down	5.57E-273	putative GID1-like gibberellin receptor
Solyc02g037500.1.1	-1.053140264	Down	1.03E-20	probable acyl-activating enzyme 6-like
Solyc11g072890.1.1	-1.049009276	Down	1.52E-25	uncharacterized protein LOC101257533
Solyc01g067670.2.1	-1.006349048	Down	6.70E-31	tubby-like F-box protein 6-like

Supplementary Table 2. Oligonucleotide primers used in this study.

Gene	Forward primer (5' to 3')	Reverse primer (5' to 3')	Application
SlMSI1-GFP	CGGGATCCATGGGGAAAGACGAAG	CGGGATCCAGGGCCTTTGGGGGC	**Construction of *pBIN-SlMSI1-GFP, pCXSN-RIN* and Screened for transgenic plants**
NPT2	GCTTGACTGCAGAACTACCG	TGAATACAAATTATTGGTGGGAAA	
antisenseSlMSI1	GCTGGCCAAATCATTTGCCAATTG	GATACTAATATAGCCCTTAGGAG	
RIN	CATGGGTAGAGGGAAAGTAGAA	TCATGTGTTGATGGTGCTTATAG	
35S	GACGGCACAATCCCACTATCC		
SlMSI1	CCACAAAACCCAAACCCTCAC	AAGGCCATTCAAGCGCGTGA	**qRT-PCR**
RIN	AAACATCATGGCATTGTGGTGAGC	ATGGTGCTGCATTTTCGGGTTGTA	
CNR	GCCAAATCAAGCAATGATGA	TCGCAACCATACAGACCATT	
TDR4	CGACAACACATTTGACGAAACC	GGGTGTGGAGTAGTGTTTCTCTG	
AP2a	CCAACAACTTCAACTTCTC	CGATATTTGGTTGCAAGTGAC	
NOR	CCCAAAATTTAATCATAATAC	CCGTACTTTCCATAAAGAAATC	
ACS2	AAGCTTAACGTCTCGCCTGG	CCACCCTGGCTCTTGACATT	
ACS4	CATGTCTCCTAATTCTCCAATTC	GGGCCCGTGCTTTAGCACGAGC	
MAN4	CAAAAATAGATTGAAGGCTATAG	CATGTATACAAAGTATTTATAC	
TBG4	CAAGAAGCACTCCACAGATGTGG	CCATTTTGATGAATCTAACAAG	
E4	CAGCAGATCTTTGAATGTCATAG	CGACAACTAGTTATTCCATCC	
E8	CCAGTAAGTGGTGTTACACCTC	GGGCAATACCCTTTAAGAAAAC	
PG	CTTTCGTCTAAATATCTCTCATC	GGTATGGTTTTTAAACTTGTC	
DDTFR18	AGTACCCAATTCTTATATCGTG	GGTTTCAATCCATACTCTTG	
AP3	GTAGTAATTTTCACCTTATTG	GGTTTTAACACACTGGATTAAGC	
PI	GATTATGGGCAAATGCCTTTTG	GAAACATAGATATAGGTAGATAG	
ACTIN	GTCCTCTTCC AGCCATCCAT	ACCACTGAGCACAATGTTACCG	
